# A dual-responsive NIR fluorescent probe for GSH and viscosity: applications in models of cellular inflammation and apoptosis

**DOI:** 10.1039/d5ra06464c

**Published:** 2025-12-12

**Authors:** Sha Li, Weize Xing, Wei Wen, Miao Yan, Haixian Ren

**Affiliations:** a Department of Chemistry, Xinzhou Normal University Xinzhou 034000 China lisaa0612@163.com

## Abstract

Cancer remains a leading cause of global death. Because high levels of glutathione (GSH) and increased intracellular viscosity are known as key biomarkers linked to cancer cells, we developed a dual-responsive near-infrared (NIR) fluorescent probe, NIR-NOS, for simultaneous detection of mitochondrial GSH and viscosity. NIR-NOS uses NOS-Br as the GSH-responsive component and a C=C bond within the NIR-1 part as the viscosity-sensitive element, with its fluorescence enhanced through the restriction of intramolecular rotation. When exposed to GSH, breaking the ether bond causes a significant fluorescence turn-on. At the same time, increased viscosity limits the rotation of the C=C bond, boosting the fluorescence at 640 nm by about 50 times. Spectroscopic tests showed excellent selectivity for GSH over other substances, with a detection limit of 0.44 µM. Importantly, NIR-NOS shows low toxicity and effectively targets mitochondria. This probe allows for visualization of both natural and induced changes in mitochondrial GSH, as well as real-time observation of intracellular viscosity shifts, demonstrated in studies using nystatin (Nys) and lipopolysaccharide (LPS) to alter these factors. Additionally, because of its mitochondrial targeting and dual-response function, NIR-NOS enables real-time tracking of key mitochondrial activities, such as vesicle formation and programmed cell death triggered by Nys. This offers deeper understanding of mitochondrial redox regulation and environmental changes. Overall, this study introduces NIR-NOS, a thoughtfully designed, multifunctional NIR fluorescent probe with great potential for advanced cancer imaging and live, *in situ* tracking of interconnected mitochondrial markers.

## Abstract 2

Cancer remains a leading cause of global death.

## Graphical abstract

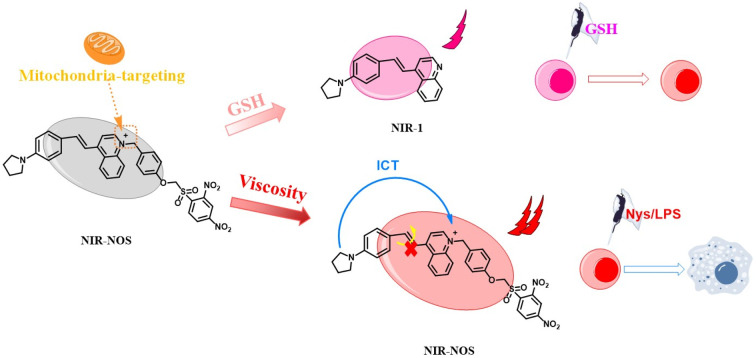

## 1. Introduction

Cancer remains one of the leading causes of death worldwide. It mainly results from mutations in proto-oncogenes and tumor suppressor genes in normal cells, caused by various carcinogenic stimuli, including physical, chemical, and viral agents. These genetic alterations pose a serious threat to global health and survival.^1–5^ Consequently, there is an increasing demand for advanced real-time diagnostic methods that can specifically and sensitively detect disease-related biomarkers and accurately locate pathological tissues. Among these methods, fluorescent probes—particularly those active in the near-infrared (NIR) region—are highly attractive due to their rapid response, high sensitivity, and excellent biocompatibility. Developing NIR fluorescent probes with high specificity and sensitivity for early cancer detection has become a crucial and valuable area of research.^6–12^ Considering the differences in microenvironmental features and levels of bioactive molecules between cancerous and healthy cells, it is vital to develop probes tailored to specific biomarkers. This enables precise detection and *in situ* visualization of tumor locations.

Glutathione (GSH) plays a vital role in maintaining mitochondrial redox balance,^13^ and its intracellular level is an important physiological marker for evaluating organism health.^14–17^ Furthermore, intracellular viscosity significantly influences the movement of biomolecules, which affects many cellular functions and interactions within the microenvironment.^18–22^ The development and progression of malignant tumors are often marked by changes in intracellular components, especially variations in the amount and physicochemical properties of substances like GSH and viscosity.^23–26^ Notably, compared to normal cells, tumor cells often show higher GSH levels and increased viscosity, making these features valuable biomarkers for cancer diagnosis and monitoring.^27–30^ However, most current probes only detect a single biomarker. To overcome this limitation, there is an urgent need to develop a fluorescent probe capable of responding to both GSH and viscosity, enabling highly sensitive imaging of cancer cells and supporting comprehensive tumor diagnosis and treatment.^31^

Considering the high levels of GSH and intracellular viscosity in cancer cells, we designed a dual-responsive NIR fluorescent probe, NIR-NOS, capable of detecting mitochondrial GSH and viscosity. NIR-NOS has a D–π–A structure that allows free intramolecular rotation, making it highly sensitive to viscosity. The GSH-responsive component, NOS-Br, connects to the NIR-1 fluorophore and modifies its ICT properties by suppressing ICT when GSH is absent. When GSH reacts with the probe, the ether bond breaks, releasing NIR-1. This reactivates the ICT process, boosting fluorescence. Additionally, quaternary ammonium (N^+^) groups help target mitochondria efficiently. During mitochondrial viscosity monitoring, we observed the formation of mitochondrial vesicles. Importantly, both GSH levels and viscosity increased significantly during inflammation and apoptosis, demonstrating NIR-NOS's potential as a useful tool for early cancer detection and studying related cellular mechanisms.

## 2. Experimental section

### 2.1. Synthesis of NIR-1

4-(1-Pyrrolidino)benzaldehyde (88 mg, 0.5 mmol) and 4-methylquinoline (172 mg, 1.2 mmol) were added to 20 mL ethanol, placed in a 50 mL round-bottom flask, and two drops of piperidine were added. The mixture was heated to 80 °C, stirred for 12 hours, then cooled to room temperature. After removing the solvent, the mixture was purified by column chromatography using DCM/MeOH (10/1, v/v), yielding a dark red solid (57 mg, 65%). ^1^H NMR (600 MHz, DMSO-d6) *δ* 8.95 (dd, *J* = 28.0, 4.7 Hz, 1H), 8.24 (dd, *J* = 31.6, 8.0 Hz, 3H), 7.78 (dd, *J* = 9.9, 7.5, 2.5 Hz, 1H), 7.71–7.51 (m, 5H), 7.40–7.26 (m, 1H), 6.98 (d, *J* = 8.5 Hz, 1H), 3.45–2.67 (m, 4H), 1.70 (dq, *J* = 34.2, 5.9 Hz, 4H). HR-MS [NIR-1 + Na]^+^: *m*/*z* calcd 323.15242, found 323.15250 (Fig. S1 and S2).

### 2.2. Synthesis of NIR-NOS

**Scheme 1:**
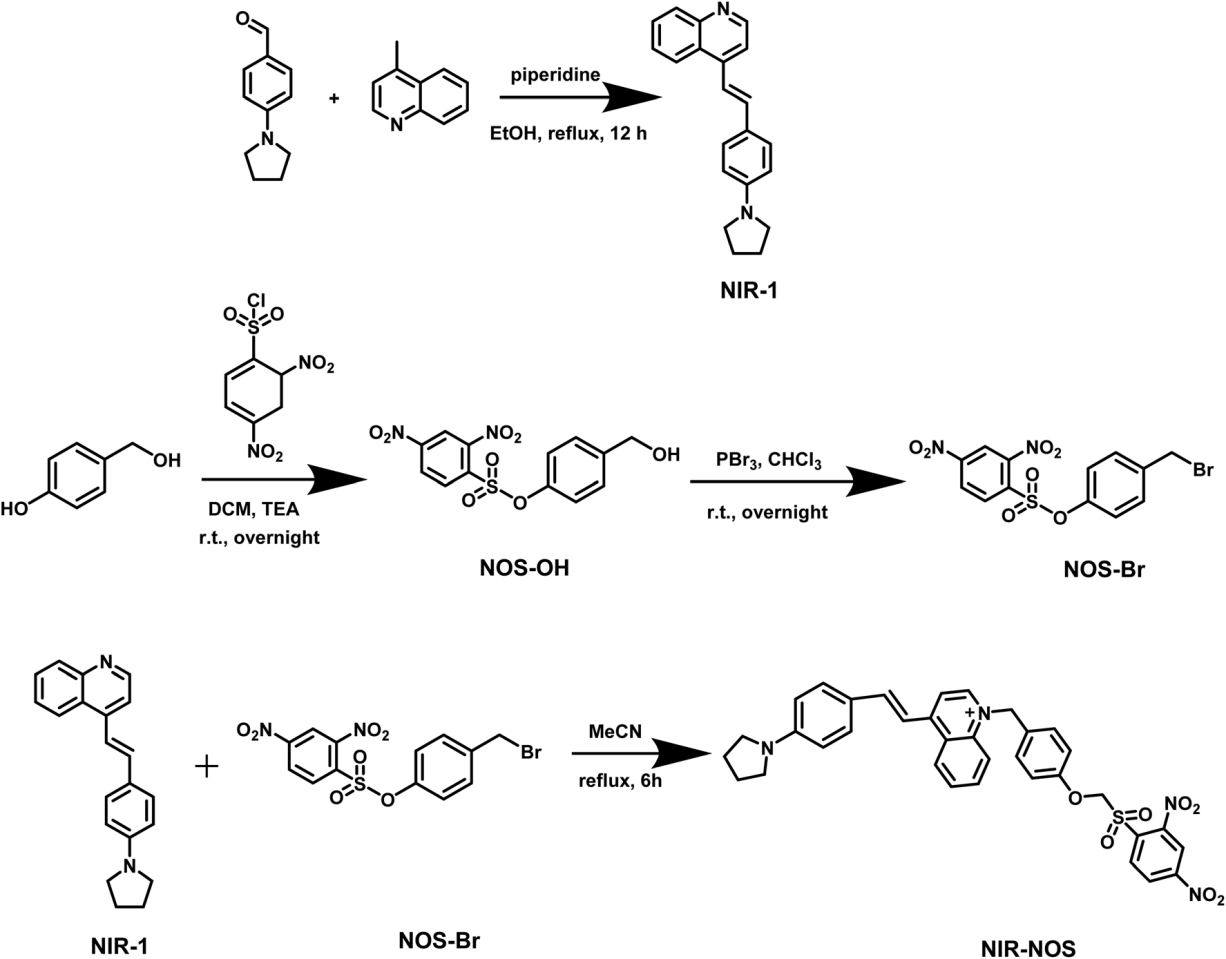
Experimental route of probe NIR-NOS.

A solution containing NIR-1 (150 mg, 0.42 mmol) and NOS-Br (147 mg, 0.42 mmol) in acetonitrile (10 mL) was heated under reflux with continuous stirring. After the reaction, the mixture was evaporated under reduced pressure and purified to obtain NIR-NOS (45 mg, 30%). ^1^H NMR (600 MHz, DMSO-d6) *δ* 8.85 (s, 1H), 8.71 (s, 3H), 8.37–8.31 (m, 4H), 8.17 (d, *J* = 8.4 Hz, 1H), 8.05 (d, *J* = 8.4 Hz, 1H), 7.97–7.93 (m, 3H), 7.85–7.82 (m, 1H), 7.72–7.69 (m, 1H), 7.65–7.61 (m, 1H), 7.50 (d, *J* = 4.5 Hz, 4H), 7.22 (d, *J* = 9.3 Hz, 4H), 2.75 (s, 3H), 1.24 (s, 4H). ^13^C NMR (151 MHz, DMSO-d6) *δ* 191.38, 187.47, 182.50, 174.44, 171.81, 167.78, 158.88, 149.22, 137.87, 137.00, 133.72, 131.20, 130.50, 129.77, 129.73, 129.05, 128.62, 128.17, 127.35, 125.02, 122.68, 122.67, 122.53, 120.67, 106.22, 98.39, 81.53, 57.73, 50.58, 29.57, 24.15, 21.97, 17.97, 16.02, 9.47. HR-MS [NIR-NOS + Na]^+^: *m*/*z* calcd 674.18057, found 674.18080 (Scheme 1).

## 3. Results and discussion

### 3.1. Reaction mechanism

**Scheme 2:**
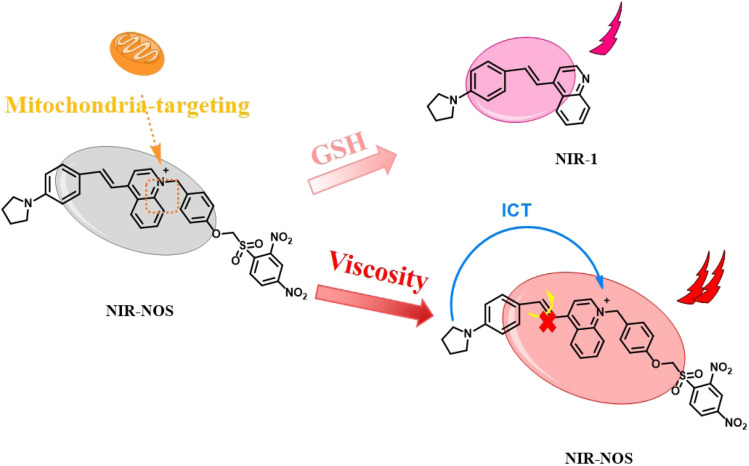
The response mechanism of NIR-NOS.

The reaction mechanism was further confirmed through HRMS tests. HR-MS [NIR-NOS + Na]^+^: *m*/*z* calcd 674.18057, found 674.18080; [NIR-1 + Na]^+^: *m*/*z* calcd 323.15242, found 323.15250 (Fig. S2). NIR-NOS contains luminescent groups based on a π-extended quinoline ring (NIR-1) combined with NOS-Br recognition groups, arranged within the D–π–A framework, and shows unique viscosity response properties. The glutathione-sensitive NOS-Br component interacts with the NIR-1 chromophore, effectively regulating intramolecular charge transfer kinetics and increasing luminescence output *via* a fluorescence recovery mechanism. As a result, NIR-NOS initially releases NIR-1 through ether bond cleavage by GSH. NIR-1 then emits weak red fluorescence due to reactivation of the ICT process. Later, due to the inhibition of intramolecular twisted charge transfer (TICT), NIR-NOS exhibits intense red fluorescence at high viscosity (Scheme 2).

### 3.2. The spectrum of NIR-NOS

**Fig. 1:**
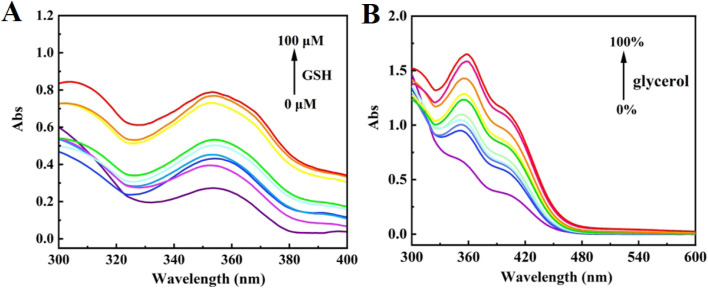
(A) Absorption spectra of NIR-NOS (10 µM) to GSH (0–100 µM) in Hepes (50% MeOH, pH 7.4); (B) absorption spectra of NIR-NOS (10 µM) to viscosity in Hepes/glycerol solutions (fG = 0–99%).

First, the fluorescence spectra of NIR-NOS in various organic solvents were examined. As shown in Fig. S3, MeOH was chosen as the solvent for spectral testing because of its excellent spectral performance in water-soluble solvents, good water solubility, and better compatibility with biological research systems. Next, the UV-visible absorption spectral changes of NIR-NOS in Hepes buffer (50% MeOH, v/v) with different GSH concentrations and solvents of varying viscosities were studied. As shown in Fig. 1A, NIR-NOS (10 µM) exhibited a maximum absorption peak at 356 nm. The absorption peak at 356 nm increased gradually with rising GSH concentrations. In Fig. 1B, the absorption peak at 362 nm increased as viscosity increased.

**Fig. 2:**
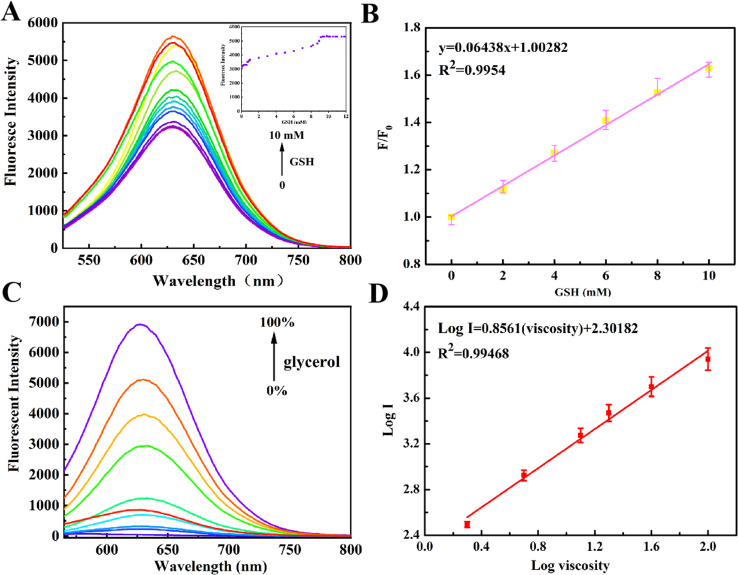
(A) Fluorescence response of NIR-NOS (10 µM) to GSH (0–10 mM) in Hepes (50% MeOH, pH 7.4, the illustration shows a scatter plot of the fluorescence intensity at 640 nm with the fluorescence intensity ratio varying with GSH (0–10 mM)); (B) linear relationship between fluorescent intensity and [GSH] (0–10 mM); (C) fluorescence response of NIR-NOS (10 µM) to viscosity in Hepes/glycerol solutions (fG = 0–99%); (D) linear relationship between log *F* and log *η*.

Further experiments examined NIR-NOS fluorescence under different GSH levels and viscosities. When excited at 356 nm, NIR-NOS displayed weak fluorescence. However, as GSH was gradually added, a significant increase in fluorescence emission at 640 nm was observed (Fig. 2A). In Fig. 2A, the illustration shows a scatter plot of fluorescence intensity at 640 nm with GSH concentrations ranging from 0 to 10 mM. We also recorded fluorescence spectra after adding GSH (0–10 mM), noting that the fluorescence intensity steadily increased with higher GSH levels. A strong linear relationship was established between fluorescence signal and GSH concentration (Fig. S4), as indicated by the regression equation *y* = 33 182.43601 + 19.59576*x*, with an excellent correlation coefficient (*R*^2^ = 0.99433) and a detection limit of 0.44 µM, surpassing many other reported fluorescent probes (the data are the average values ± standard deviation (*n* = 3) of three independent experiments).

As shown in Fig. 2C, the fluorescence of NIR-NOS was further examined in relation to viscosity. As viscosity increased, the fluorescence intensity significantly rose to 50 times that of the pure probe. A strong linear relationship was observed between log(*I*) and log(*η*) for NIR-NOS (log(*I*) = 0.8561 log(*η*) + 2.30182, *R*2 = 0.9789) (Fig. 2D).

To verify the specificity of the NIR-NOS viscosity sensor and to rule out potential interference from GSH, we performed an orthogonal comparison experiment between the viscosity response of NIR-NOS and the molecular rotor T-N. As shown in Fig. S5, in the glycerol-water mixture ranging from 0% to 99%, NIR-NOS showed a significant fluorescence increase depending on viscosity. Importantly, in the system containing 100 µM GSH, its fluorescence-viscosity response curve was nearly identical to that of the system without GSH. This clearly demonstrates that GSH does not interfere with NIR-NOS's ability to sense viscosity within physiological concentration ranges, confirming its high specificity.

**Fig. 3:**
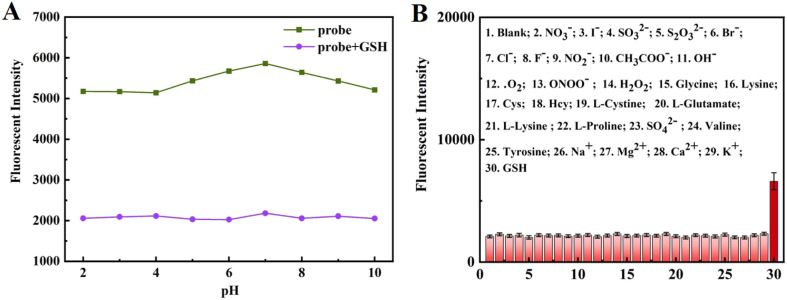
(A) The fluorescence changes of NIR-NOS (10 µM) in the absence and presence of 100 µM GSH in MeOH/Hepes (1/1, v/v, pH 7.4) at different pH values. (B) The fluorescence intensity of NIR-NOS (10 µM) with GSH and other amino acids (10 equiv.).

Fig. 3A shows the fluorescence responses of NIR-NOS when exposed to GSH across different pH levels (2–10). Notably, the highest fluorescence intensity at 640 nm occurs at physiological pH (7.4), indicating the probe works best in neutral environments. To further evaluate its suitability for biological applications, we tested its photostability. As shown in Fig. S6, both the free probe and the probe-GSH complex maintained stable fluorescence over 30 min, demonstrating high temporal stability.

We explored the specificity of NIR-NOS at the above pH and reaction time. As can be seen in Fig. 3B, in the presence of different cations, anions and various amino acids (NO_3_^−^; I^−^; SO_3_^2−^; S_2_O_3_^2−^; Br^−^; Cl^−^; F^−^; NO_2_^−^; CH_3_COO^−^; OH^−^; ˙O_2_; ONOO^−^; H_2_O_2_; glycine; lysine; Cys; Hcy; l-Cys; l-Glu; l-Lys; l-Pro; SO_4_^2−^; valine; tyrosine; Na^+^; Mg^2+^; Ca^2+^; K^+^; GSH) (each at a concentration of 100 µM), only GSH resulted in a significant enhancement of the fluorescence intensity of the NIR-NOS, indicating that the probe has a high specificity for GSH.

### 3.3. Theoretical calculations

**Fig. 4:**
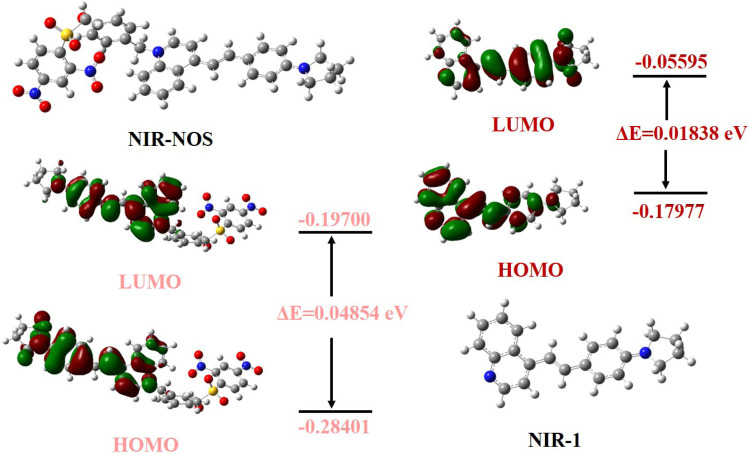
Theoretical calculations of NIR-NOS and NIR-1.

As shown in Fig. 4, the electronic structures of NIR-NOS and its product NIR-1 were modeled using density functional theory (DFT) calculations. The frontier molecular orbital analysis indicated that the electron density of both the highest occupied molecular orbital (HOMO) and the lowest unoccupied molecular orbital (LUMO) in NIR-NOS is mainly localized on the NOS-Br group. In contrast, the electron clouds of the HOMO and LUMO in NIR-1 are evenly spread across the entire NIR-1 molecule. This clear difference in electron density distribution suggests that breaking the ether bond in NIR-NOS when reacting with GSH releases the NIR-1 fluorophore, restoring its natural ICT property. The renewed ICT process causes the observed red-shifted fluorescence emission.

### 3.4. Imaging of GSH in HeLa cells

To demonstrate the *in vivo* applicability of NIR-NOS, we assessed its cytotoxicity using the CCK-8 assay (Fig. S7). HeLa cells incubated with 10 µM NIR-NOS alone for 12 hours at 37 °C showed an approximately 80% survival rate, indicating that NIR-NOS has low cytotoxicity.

**Fig. 5:**
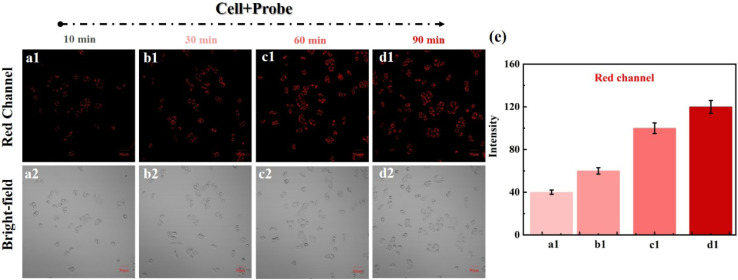
Cells incubated with probe NIR-NOS for 10, 30, 60, and 90 min, respectively. (a1–d1) Red channel (*λ*_ex_/*λ*_em_ = 488/620–680 nm). (a2–d2) Bright field. (e) The fluorescence intensity of a1–d1. Scale bar = 50 µm.

Based on the excellent performance of the above spectroscopic tests, we conducted confocal imaging of HeLa cells. To evaluate NIR-NOS for imaging cellular GSH, HeLa cells were incubated with NIR-NOS. The results showed that the fluorescence in the red channel gradually increased during incubation as NIR-NOS reacted with intracellular GSH to produce strong red fluorescence (NIR-1, Fig. 5).

**Fig. 6:**
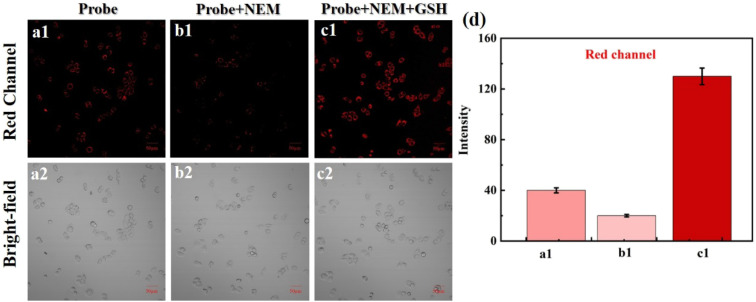
HeLa cells images: (a1–a2) cells with NIR-NOS. (b1–b2) After pretreated with NEM, cells with NIR-NOS, respectively; (c1–c2): after pretreated with NEM, cells with GSH and NIR-NOS, respectively. (d) The relative fluorescence intensities of (a1–c1). *λ*_ex_/*λ*_em_ = 488/620–680 nm, scale bar = 50 µm.

Subsequently, we tested the use of NIR-NOS for imaging cellular exogenous GSH (Fig. 6). The HeLa cells were first stained with NIR-NOS to observe fluorescence. Then, in the second group of HeLa cells, the cellular intrinsic biothiols were removed using NEM, followed by NIR-NOS staining; no significant fluorescence was seen in these cells. In the final group, we incubated the cells with NEM to remove their intrinsic biothiols, then added GSH, which caused an increase in red fluorescence in the cells.

### 3.5. Imaging of viscosity in HeLa cells

**Fig. 7:**
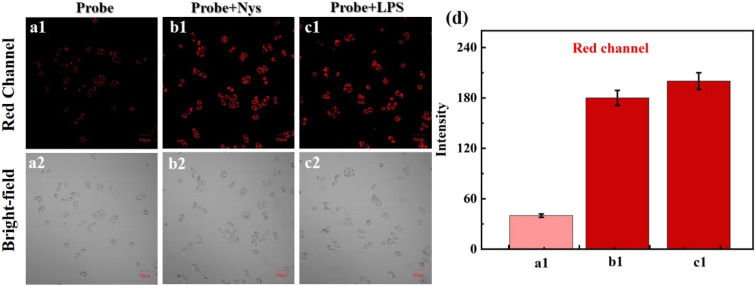
The viscosity variation of NIR-NOS imaging. (a1–a2) Only added NIR-NOS. (b1–b2) Incubated with Nys and then stained with NIR-NOS. (c1–c2) Incubated with LPS and then added the NIR-NOS. (d) The relative fluorescence intensities of (a1–c1). *λ*_ex_/*λ*_em_ = 488/620–680 nm, scale bar = 50 µm.

To verify NIR-NOS's ability to image changes in intracellular viscosity, we stimulated HeLa cells with nystatin (Nys). As shown in Fig. 7, cells incubated only with NIR-NOS exhibited weak red fluorescence. In contrast, after stimulation with mycophenolate, cell fluorescence increased significantly (Fig. 7b1). Interestingly, some cells formed blisters in response to mycophenolate stimulation (Fig. 7b2), which caught our attention. Since lipopolysaccharide (LPS) induces cellular inflammation, and these inflamed cells can lead to increased viscosity, we examined the dynamic changes in mitochondrial viscosity and GSH, along with their potential regulatory roles, during LPS-induced inflammation and oxidative stress responses using NIR-NOS.^32–36^ Similar to previous experiments, under LPS stimulation, the red channel fluorescence gradually increased compared to the baseline, indicating an increase in the viscosity of inflammatory cells (Fig. 7c1). These results suggest that NIR-NOS can effectively detect changes in mitochondrial viscosity.

### 3.6. Colocalization experiments in living cells

**Fig. 8:**
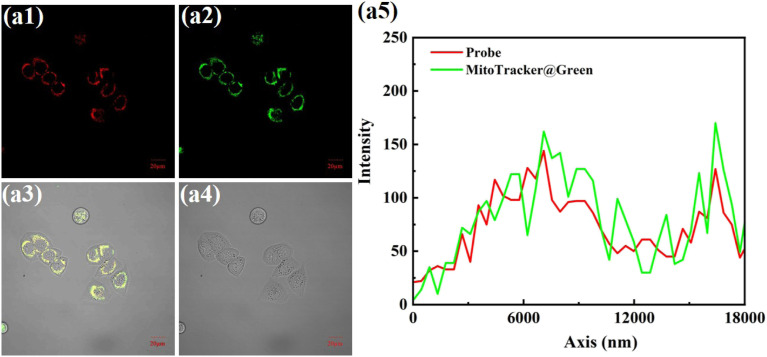
HeLa cells incubated with NIR-NOS (10 µM) and MTG (50 nM) for 30 min respectively. (a1) Red channel imaging (*λ*_ex_ = 561 nm, *λ*_em_ = 566–718 nm); (a2) green channel image (*λ*_ex_ = 488 nm, *λ*_em_ = 493–596 nm); (a3) combined image of (a1) and (a2); (a4) Bright-field image; (a5) the intensity of the linear ROI. Scale bar = 20 µm.

To confirm mitochondrial localization, we stained HeLa cells with MitoTracker Green. In Fig. 8, the green and red fluorescence signals overlapped well, with a Pearson's coefficient of 0.9.

We also compared the fluorescence properties of NIR-NOS with other similar fluorescent probes for detecting GSH (Table S1). The results show that NIR-NOS effectively combines multiple excellent features such as dual-response functionality, near-infrared emission, low detection limit, and specific mitochondrial targeting into a single probe molecule. This well-rounded and balanced design allows it to perform high-fidelity imaging of the mitochondrial microenvironment related to diseases like cancer at the cellular level, demonstrating its potential for practical application.

## 4. Conclusion

In summary, we developed a rationally engineered dual-responsive NIR fluorescent probe, NIR-NOS, capable of simultaneously monitoring mitochondrial GSH levels and viscosity changes for precise biomarker imaging in cancer cells. The probe uses a modular design: the NOS-Br component acts as a specific GSH recognition unit through thiol-mediated ether bond cleavage, while the rotatable C=C bond within the NIR-1 fluorophore functions as a molecular rotor for viscosity detection. When GSH levels rise, the ether bond is cleaved, releasing the NIR-1 fluorophore. Increased microenvironmental viscosity then restricts the intramolecular rotation of the C=C bond, boosting fluorescence emission through the restriction of intramolecular rotation mechanism. Importantly, NIR-NOS shows excellent photostability under physiological conditions and high resistance to interference from reactive oxygen species, enabling accurate imaging of GSH dynamics in tumor cells. The probe's effectiveness has been confirmed through real-time tracking of viscosity changes caused by Nys and LPS. NIR-NOS presents great potential as a targeted diagnostic tool for cancer detection based on mitochondrial microenvironment profiling.

## Conflicts of interest

There are no conflicts to declare.

## Supplementary Material

RA-015-D5RA06464C-s001

## Data Availability

The data supporting this article have been included as part of the supplementary information (SI). Supplementary information is available. See DOI: https://doi.org/10.1039/d5ra06464c.
