# Supplementary material for: A dual-responsive NIR fluorescent probe for GSH and viscosity: applications in models of cellular inflammation and apoptosis
Source: RSC Adv. 2025 Dec 12;15(57):49516–24. doi: 10.1039/d5ra06464c (PMC12699297; doi:10.1039/d5ra06464c)
Supplement: RA-015-D5RA06464C-s001 [file RA-015-D5RA06464C-s001.pdf]

Supplementary Information (SI) for RSC Advances.
This journal is © The Royal Society of Chemistry 2025

A Dual-Responsive NIR Fluorescent Probe for GSH and Viscosity: Applications in Models of Cellular Inflammation and Apoptosis

Sha Li [a]*, Weize Xing [a], Wei Wen [a], Miao Yan [a], Haixian Ren [a]
[a]Department of Chemistry, Xinzhou Normal University, Xinzhou 034000, China

1.Experimental Section

1.1 Materials and instruments

The drugs and solvents used were all of analytical purity level. The experimental reagents were all purchased from Aladdin Pharmaceuticals and did not require further purification. All chemicals from Aladdin were used without further purification. Fluorescence spectra were carried out a HITACHI F-7000 spectrophotometer. UV-visible spectra were recorded with a HITACHI U-3900 spectrophotometer. NMR spectra were recorded on a JBruker AVANCE-600MHz spectrometer and chemical shifts were referenced relative to tetramethylsilane. Mass data (ESI) were obtained by an AB Triple TOF 5600plus System (AB SCIEX, Framingham, USA). The final bioimaging application were measured by the Zeiss LSM880 Airyscan confocal laser scanning microscope.

1.2 Synthesis of NOS-Br

NBD-Cl (99.8 mg, 0.5 mmol) was dissolved in DCM (15 mL). After stirring in ice bath for 5 min, 4-hydroxybenzyl alcohol (62.07 mg, 0.5 mmol) dissolved in 10 mL DCM was added in batches. Then 200 μL triethylamine was added and the reaction was complete for 8 h. After decompression, the solvent was removed and purified by silica gel column chromatography to obtain the **NOS-OH** (60 mg, 60%).

NBD-OH (0.718 g, 2.5mmol) was dissolved in chloroform (20 mL) in a nitrogen atmosphere, and phosphorus tribromide (0.8 g, 3 mmol) was added in drops at room temperature. The mixture was stirred overnight at room temperature. After the reaction was complete, the solvent was removed by decompression and purified by silica gel column chromatography to obtain the **NOS-Br** (0.65 g, 90%).

E-mail address: lisaa0612@163.com

1.3 Solution preparation and optical measurement

Dissolve the probe (2 mM) in DMSO as a stock solution. GSH (20 mM) solution was prepared by dissolved glutathione in deionized water. Add the fixed concentration **NIR-NOS** probes to solutions with different glycerol ratios respectively. Vortex mix them, let them balance, and then conduct the fluorescence response test.

Stock solutions (100 mM) of NO_3^-; I^-; SO_3^{2-}; $S_2O_3^{2-}$; Br^-; Cl^-; F^-; NO_2^-; CH_3COO^-; OH^-; $\cdot O_2$; $ONOO^-$; H_2O_2; Glycine; Lysine; Cys; Hcy; L-Cys; L-Glu; L-Lys; L-Pro; SO_4^{2-}; Valine; Tyrosine; Na^+; Mg^{2+}; Ca^{2+}; K^+; GSH, were prepared by direct dissolution of deionized water. UV-vis and fluorescence spectra were detected in Hepes (50% MeOH, pH 7.4) solutions. Fluorescence measurements were carried out with a slit width of 5 nm/2 nm (λ_{ex}=356 nm).

1.4 Cell imaging experiments

The HeLa cells were cultured in DMEM medium (12% FBS and 1% antibiotics) in an incubator at 37°C and 5% CO_2. The cells were laid on 6-well plates and incubated overnight. Cells were plated in 6-well plates, and cultured overnight. The cells were washed with PBS three times before being imaged with a confocal laser scanning microscope. DMSO-soluble **NIR-NOS** was added to the cell medium (2 mL) at a final concentration of 10 μM. After incubating for 15 minutes, gently wash the excess **NIR-NOS** with PBS (10mM, pH=7.4) for 3 times. Meanwhile, another portion of HeLa cells pretreated with 10 μM **NIR-NOS** was treated with 100 μM GSH and incubated at 37°C for 10 min. Before imaging, the stained HeLa cells were washed with PBS once. The **NIR-NOS** enables the red channel (λex/λem = 488/620–680 nm, scale bar = 50 μm.) recognition and fluorescence imaging on the Zeiss LSM880 Airyscan confocal laser scanning microscope. All quantitative data from cell imaging are derived from at least three independent biological replicate experiments (n≥3). The data are presented as the mean± standard deviation (Mean±SD).

2.Characterization of compounds

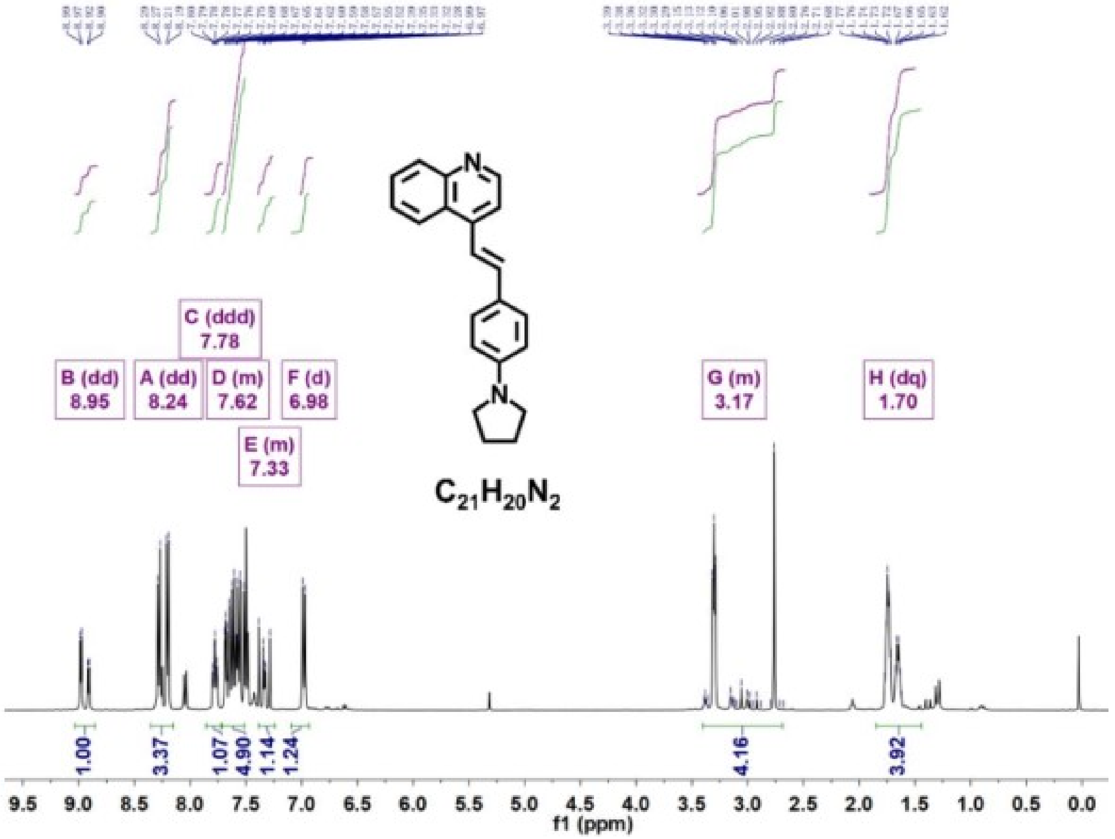

^{1}H NMR (600 MHz) of **NIR-1** in DMSO-d_6

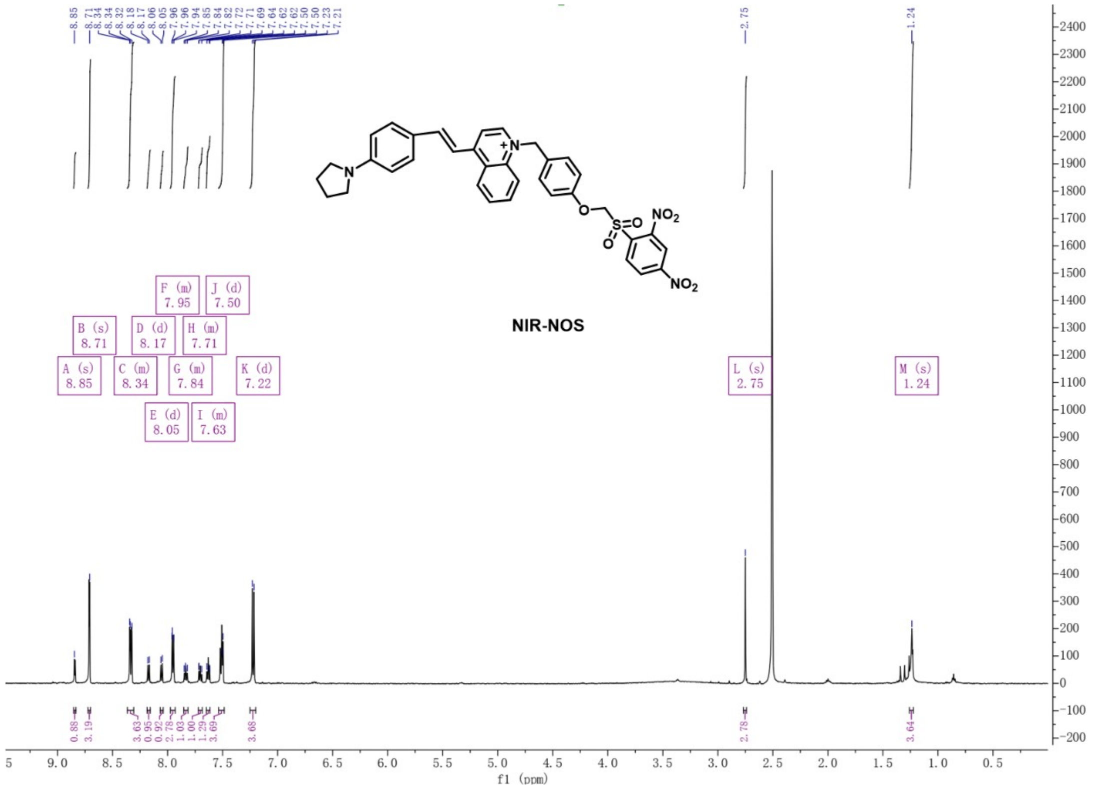

^{1}H NMR (600 MHz) of **NIR-NOS** in DMSO-d_6

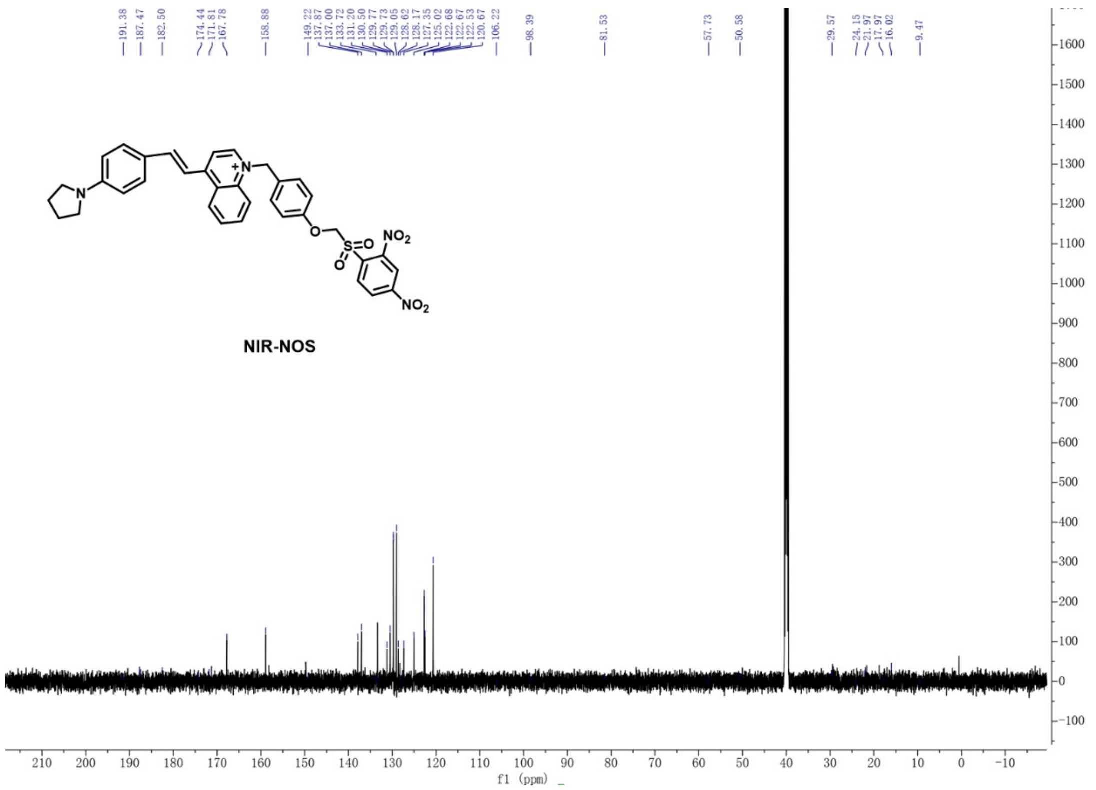

Fig. S1 ^{13}C NMR (151 MHz) of **NIR-NOS** in DMSO-d_6

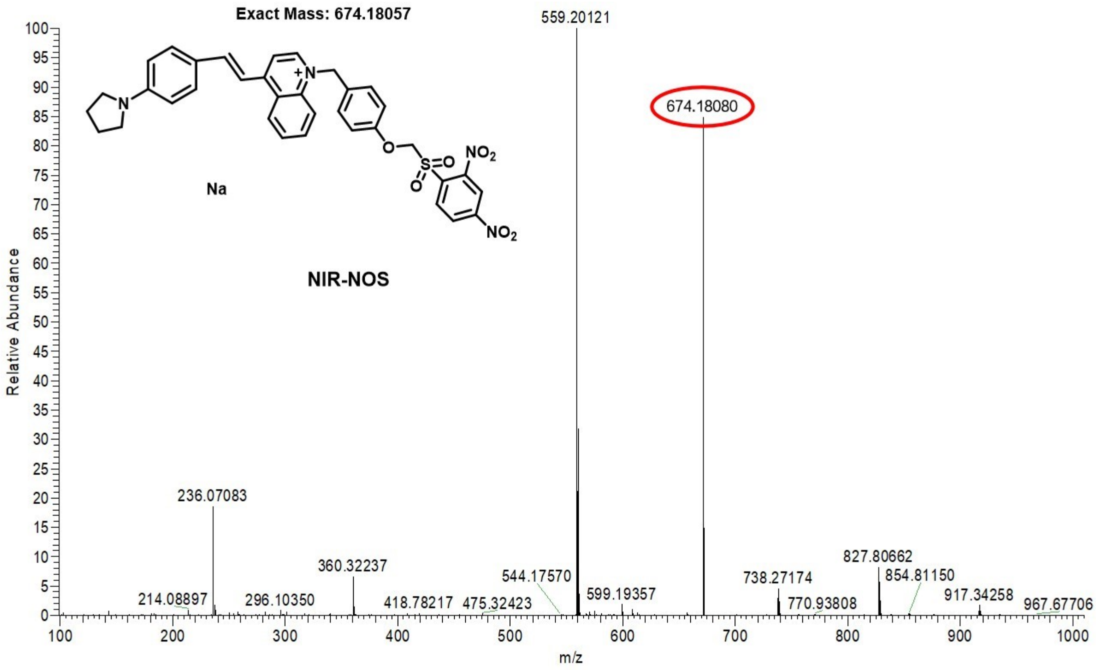

HR-MS spectra of **NIR-NOS**

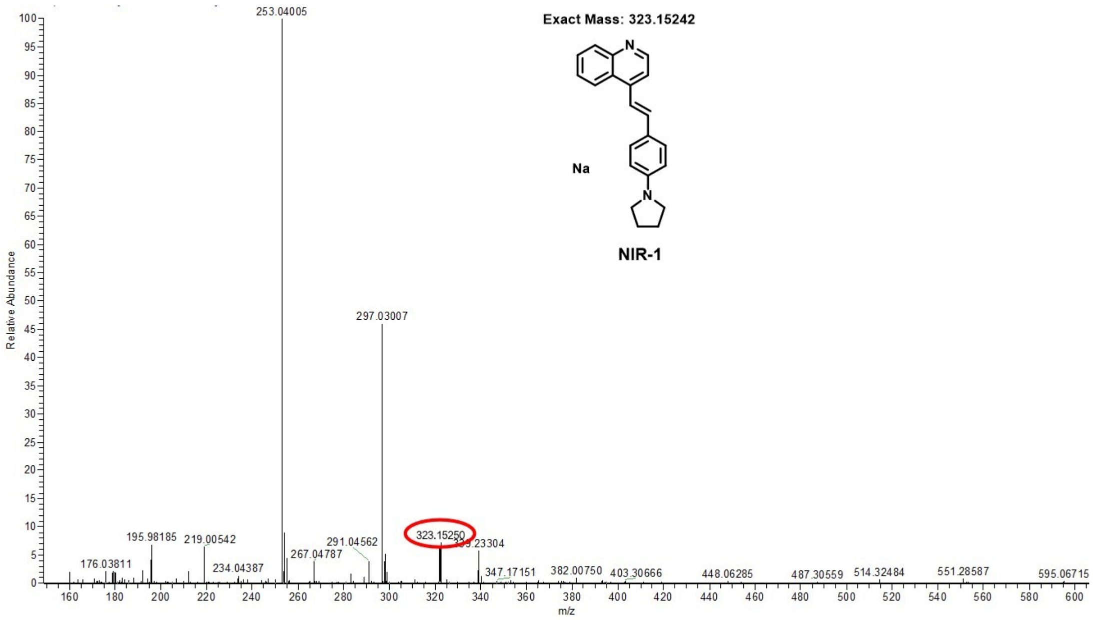

Fig. S2 HR-MS spectra of **NIR-NOS** upon addition of GSH.

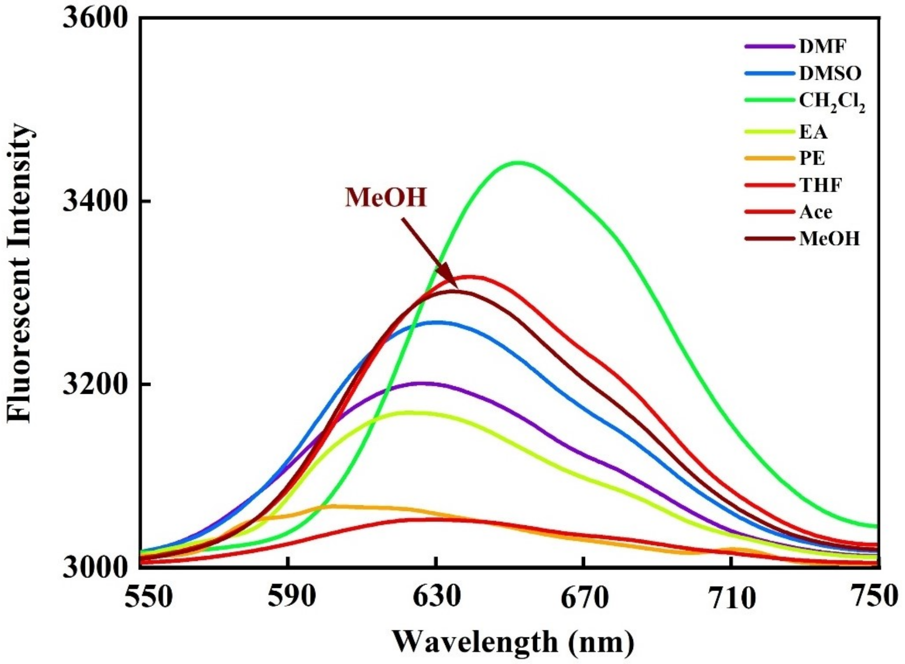

Fig. S3 The fluorescence spectra of **NIR-NOS** in different organic solvents.

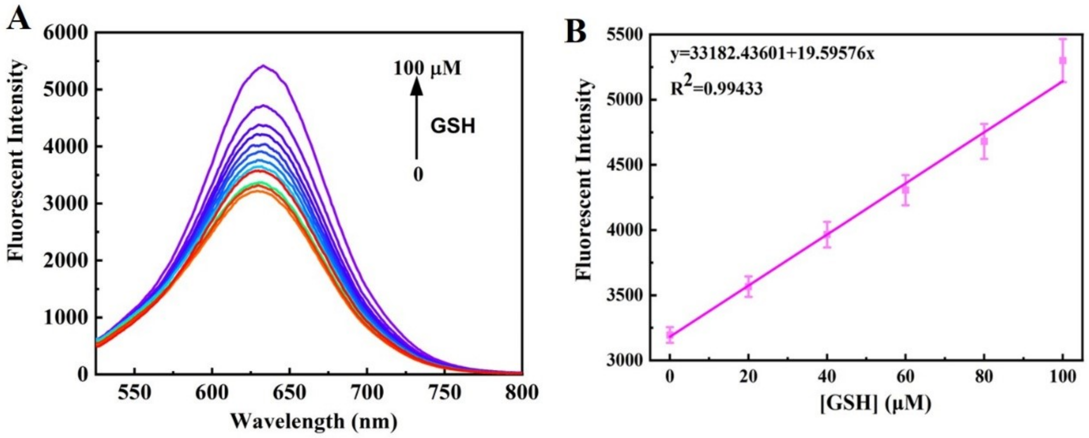

Fig. S4 Fluorescence response of **NIR-NOS** (10 μM) to GSH (0–100 μM) in Hepes (50% MeOH, pH 7.4); (B) Linear relationship between Fluorescent Intensity and [GSH] (0–100 μM).

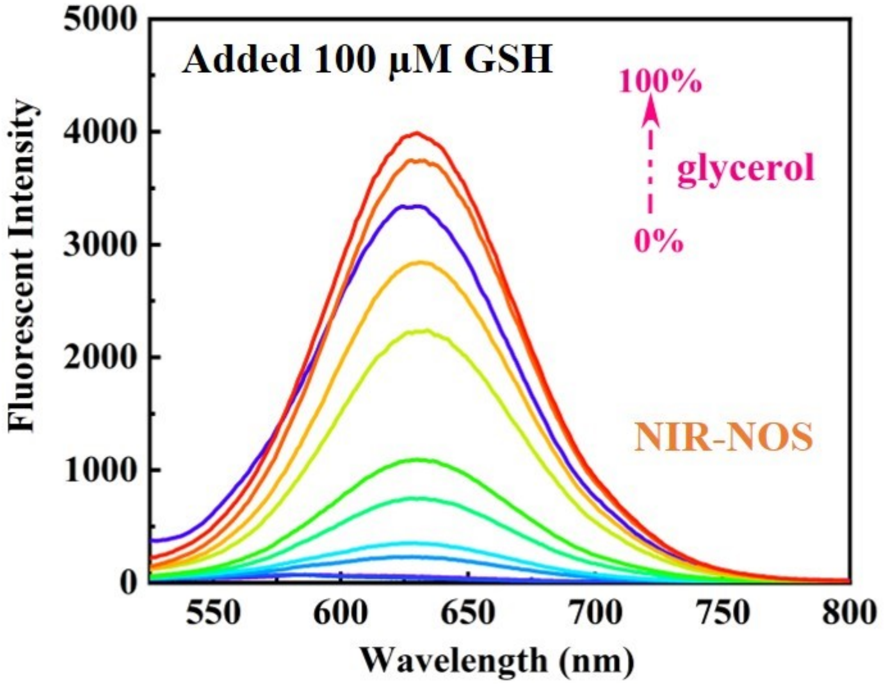

Fig. S5 The fluorescence response of **NIR-NOS** to viscosity under GSH (100 μM).

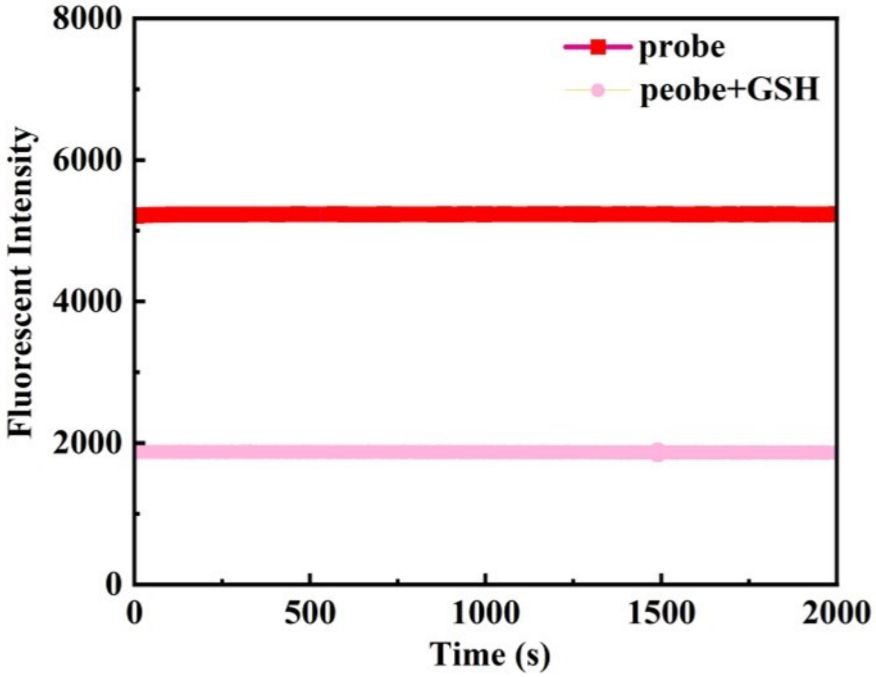

Fig. S6 Time response of probe **NIR-NOS** with GSH.

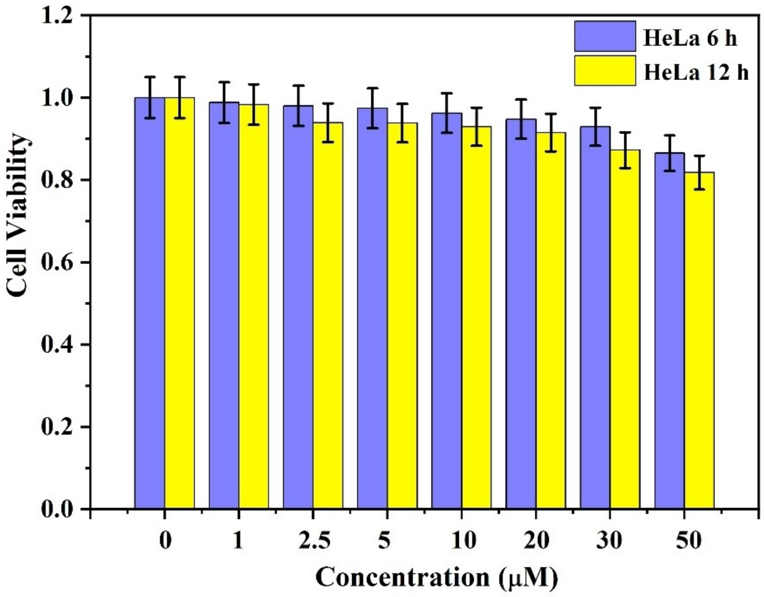

Fig. S7 Cell viability estimated by MTT assay with HeLa cells, which were cultured in the presence of 0-50 μM **NIR-NOS** for 6 h and 12 h.

Table S1 Comparison of the fluorescence properties of **NIR-NOS** and other similar fluorescent probes for the detection of GSH.

**Probe**	**Detection object**	**Fluorescence response**	**Emission wavelength**	**LOD (μM)**	**Cellular organelle targeting**	**Ref.**
1	ATP/GSH	Turn-on	467	3.47	mitochondria	**[1]**
2	Cys/Hcy/GSH	Turn-on	542	0.54	lysosome	**[11]**
3	ClO^-/GSH	Turn-off	770	7.2	-	**[14]**
4	Cys/Hcy/GSH	Turn-on	560	0.36	-	**[19]**
5	GSH	Turn-on	558	-	ER	**[20]**
6	GSH	Turn-on	460	0.95	-	**[24]**
7	H_2O_2/GSH	Turn-on	455	8.4	-	**[26]**
**8**	GSH/Viscosity	Turn-on	640	0.44	mitochondria	**This work**
